# The Prognostic Role of Lactate Concentrations after Aneurysmal Subarachnoid Hemorrhage

**DOI:** 10.3390/brainsci10121004

**Published:** 2020-12-17

**Authors:** Narcisse Ndieugnou Djangang, Pamela Ramunno, Antonio Izzi, Alessandra Garufi, Marco Menozzi, Daniela Diaferia, Lorenzo Peluso, Chiara Prezioso, Marta Talamonti, Hassane Njimi, Sophie Schuind, Jean-Louis Vincent, Jacques Creteur, Fabio Silvio Taccone, Elisa Gouvea Bogossian

**Affiliations:** 1 Department of Intensive Care, Erasme Hospital, Université Libre de Bruxelles, Route de Lennik, 808 1070 Brussels, Belgium; ndieugnou@gmail.com (N.N.D.); pamela.ramunno@ulb.ac.be (P.R.); antonio_izzi2010@libero.it (A.I.); garufi.ale@gmail.com (A.G.); marc.menoz@gmail.com (M.M.); daniend87@gmail.com (D.D.); lorenzopeluso80@gmail.com (L.P.); c.prezioso89@gmail.com (C.P.); marty.talamonti@gmail.com (M.T.); hnjimi@ulb.ac.be (H.N.); jlvincen@intensive.org (J.-L.V.); Jacques.creteur@erasme.ulb.ac.be (J.C.); ftaccone@ulb.ac.be (F.S.T.); 2 Department of Neurosurgery, Erasme Hospital, Université Libre de Bruxelles, Route de Lennik, 808 1070 Brussels, Belgium; sophie.schuind@erasme.ulb.ac.be

**Keywords:** lactate, glucose, subarachnoid hemorrhage, prognosis, biomarkers, Glasgow outcome scale, mortality

## Abstract

Blood lactate concentrations are often used to assess global tissue perfusion in critically ill patients; however, there are scarce data on lactate concentrations after subarachnoid hemorrhage (SAH). We aimed to assess the prognostic role of serial blood lactate measurements on hospital mortality and neurological outcomes at 3 months after SAH. We reviewed all SAH patients admitted to the intensive care unit from 2007 to 2019 and recorded the highest daily arterial lactate concentration for the first 6 days. Patients with no lactate concentration were excluded. Hyperlactatemia was defined as a blood lactate concentration >2.0 mmol/L. A total of 456 patients were included: 158 (35%) patients died in hospital and 209 (46%) had an unfavorable outcome (UO) at 3 months. The median highest lactate concentration was 2.7 (1.8–3.9) mmol/L. Non-survivors and patients with UO had significantly higher lactate concentrations compared to other patients. Hyperlactatemia increased the chance of dying (OR 4.19 (95% CI 2.38–7.39)) and of having UO in 3 months (OR 4.16 (95% CI 2.52–6.88)) after adjusting for confounding factors. Therefore, initial blood lactate concentrations have prognostic implications in patients with SAH; their role in conjunction with other prognostic indicators should be evaluated in prospective studies.

## 1. Introduction

Aneurysmal subarachnoid hemorrhage (aSAH) is a significant cause of morbidity and mortality and is responsible for a large economic burden worldwide [1,2]. Despite improvements in diagnostic and treatment strategies, many survivors of SAH have long-term physical and cognitive deficits, significantly impairing their quality of life [2].

SAH is a highly inflammatory condition promoting both neurological and systemic inflammation [3,4]. As a consequence of these phenomena, patients often experience sympathetic hyperactivation, also known as a “catecholamine storm” [5], which can promote myocardial dysfunction, neurogenic pulmonary edema [6], hyperglycemia [7], and hyperlactatemia [8]. Hyperglycemia and hyperlactatemia have both been extensively studied as biomarkers of disease severity in critically ill patients [9] and are important predictors of poor outcome [10,11]. In patients with SAH, most studies have focused on cerebral lactate concentrations assessed by microdialysis or jugular bulb catheters and have reported a relationship between high lactate concentrations, cerebral ischemia, and poor outcome [12,13,14]. However, the prognostic value of early systemic lactate concentrations in these patients is still poorly defined: Although some studies reported an association between early high lactate concentrations and poor outcome [15,16,17,18], others did not [19].

Moreover, most studies focused on either the first lactate measurement on admission or the highest lactate concentration in the first 24 h [15,16,17]; the evolution of lactate kinetics in the acute phase of SAH was addressed in only one study [17]. We therefore investigated the evolution of blood lactate concentrations in the first days after aSAH to determine their association with outcome. 

## 2. Materials and Methods

### 2.1. Study Design 

This study was performed in the 35-bed Department of Intensive Care at Erasme Hospital, Brussels, Belgium. The local ethics committee approved the study (P2020/049) which was conducted in accordance with the ethical standards of the Declaration of Helsinki and waived the need for informed consent due to its retrospective nature. 

### 2.2. Study Participants 

We reviewed all adult (>18 years) patients admitted to our department because of non-traumatic aSAH between August 2007 and March 2019, and who had at least one arterial lactate measurement in the first 24 h of admission. The diagnostic work-up and management of SAH patients in our unit is detailed elsewhere [20].

### 2.3. Data Collection

Demographics, pre-existing chronic diseases, and the extent of hemorrhage were collected for all patients. The following severity and prognostic scores were recorded on admission: Acute Physiology and Chronic Health Evaluation II (APACHE II) score [21], Glasgow Coma Scale (GCS) [22], World Federation of Neurological Surgeons (WFNS) score [23], and Fisher scale [24] on cerebral computed tomography (CT)-scan. Patients were classified as “poor-grade” if the WFNS score on admission was 4 or 5. We also recorded the use of vasoactive drugs, inotropic agents, mechanical ventilation, renal replacement therapy (RRT), and extracorporeal membrane oxygenation (ECMO). Neurological complications, such as delayed cerebral ischemia (DCI), intracranial hypertension (i.e., intracranial pressure (ICP) > 20 mmHg for more than 5 min requiring specific therapies), hydrocephalus (i.e., Evans index > 0.35), re-bleeding, and seizures (convulsive or non-convulsive) were also recorded.

For the first 6 days after ICU admission, we recorded the highest daily lactate and glucose concentrations, the lowest and highest mean arterial pressure and heart rate values, the cumulative daily dose of vasopressors and inotropes, and the Sequential Organ Failure Assessment (SOFA) score [25]. Lactate and glucose were available from blood gas analyses at least 5 times a day for each patient. Hyperlactatemia was defined as a lactate concentration >2 mmol/L at any time during the study period [26]. The peak lactate concentration was defined as the highest lactate value recorded during the first 6 days after admission. Hyperglycemia was defined as a blood glucose concentration >180 mg/dL at any time during the study period [27]. The peak glucose concentration was defined as the highest blood glucose value recorded during the first 6 days after admission. Finally, we recorded ICU length of stay, hospital length of stay, ICU and hospital mortality, and neurological status at 3 months. Neurological status was assessed using the Glasgow Outcome Scale [28] (GOS; from 1 = death to 5 = good recovery) and evaluated from the medical records of the neurological follow-up for the patients. A favorable neurological outcome was defined as a GOS of 4–5; unfavorable neurological outcome (UO) as a GOS of 1–3.

### 2.4. Statistical Analysis

Descriptive statistics were computed for all study variables. Categorical data are presented as numbers and percentages. Continuous data were presented as mean (±standard deviation) or median (25th–75th percentiles), according to the distribution pattern of each variable. Differences between groups were assessed using a χ-square or Fisher’s exact test for categorical variables; we used a Student’s *t*-test for normally distributed continuous variables and a Mann–Whitney test for asymmetrically distributed continuous variables. The last observation carried forward (LOCF) method was used to input data lactate in the case of missing during the first six days. We performed a linear mixed model to express the time course of lactate and glucose concentrations during the first 6 days according to the neurological outcome and hospital mortality. Binary logistic regression was performed to investigate a potential causal association between hyperlactatemia and mortality/unfavorable neurological outcome. We used a directed acyclic graph to determine cofounding variables and we adjusted the model accordingly [29]. Adjusted odds ratios (ORs) with 95% confidence intervals (CIs) were computed for all variables. The independence of errors, presence of multicollinearity, and the presence of influential outlier assumptions were checked and none of them were violated. A *p* < 0.05 was considered statistically significant. Statistical analyses were performed using IBM SPSS Statistics 26 (IBM, Somers, NY, USA).

## 3. Results

### 3.1. Study Population

A total of 497 adult patients were admitted for aSAH during the study period; 41 had no lactate measurements during the first 24 h of admission and were excluded. The remaining 456 patients were included in the final analysis. The median age of the population was 54 (IQR 46–63) years and most were female (290/456, 64%); 216 (47%) patients had poor grade SAH according to their WFNS score. The most common neurological complication was intracranial hypertension (*n* = 192, 42%). The median length of stay in the ICU was 5 (2–14) days and the median hospital length of stay was 17 (5–28) days; 158 (35%) patients died before hospital discharge and 209 (46%) had an UO. These results are shown in Table 1. 

### 3.2. Lactate and Glucose Concentrations

The median number of daily lactate values available per patient was 4 (3–6). The median peak lactate concentration was 2.7 (1.8–3.9) mmol/L and the median peak glucose concentration was 188 (157–231) mg/dL. Hyperlactatemia occurred in 234 (51%) patients in the first 24 h of admission and in 310 (68%) patients at some point during the 6-day study period. The highest daily lactate levels peaked in the first 48 of admission, as shown in Table 2. Hyperglycemia was also a frequent finding during the first 6 days of the ICU stay (*n* = 256 (56%)); only 38 (8%) of patients had known diabetes mellitus on hospital admission. 

### 3.3. Lactate Concentrations and Hospital Mortality

Non-survivors were older, had a higher APACHE score, and had more frequently a poor grade status than survivors. The occurrence of re-bleeding, hydrocephalus, DCI, and intracranial hypertension was also higher in non-survivors compared to survivors. Non-survivors had more frequently hyperlactatemia during the first 6-days after admission (139/158 (88%) vs. 171/298 (57%), *p* = 0.001)) than survivors (Table 2).

**Figure 1:**
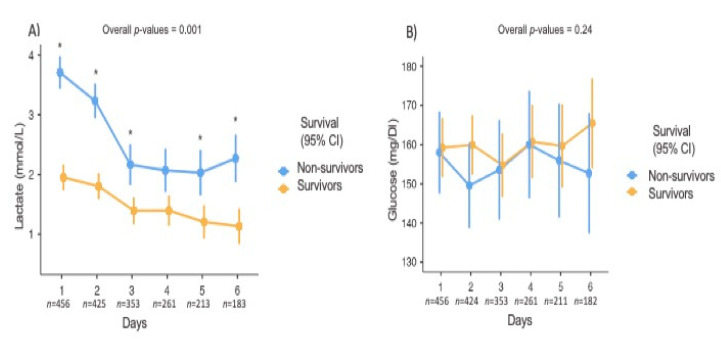
Time course of lactate (**A**) and glucose (**B**) concentrations according to in-hospital mortality. Lactate and glucose concentrations are expressed as means with 95% confidence intervals. Statistical analyses were conducted using a linear mixed model. * *p* < 0.05 obtained by a post hoc analysis using the Bonferroni method; *n* represents the number of patients included in the analysis for each day.

Figure 1 shows the time course of lactate and glucose concentrations according to hospital mortality; non-survivors had higher lactate, but not glucose, concentrations, throughout the 6-days after ICU admission than survivors (*p* = 0.001). Patients with hyperlactatemia had 3.4 times the risk of dying than patients with normal lactate levels. After adjusting for the confounders such as age, Fisher scales 3 and 4, cirrhosis, hyperglycemia, hypotension, and seizures, hyperlactatemia during the first 6-days of hospital stay was associated with an increased chance of in-hospital death (OR 4.19, 95% CI 2.38–7.39). The direct acyclic graph used to identify confounders and the logistic model that followed are presented in Supplemental Electronic Figure S1 and Table S1.

### 3.4. Lactate Concentrations and Neurological Outcome

Patients with UO were older, had a higher APACHE II score, and a lower GCS score on admission than those with a favorable outcome (Table 1). Patients with UO also had more neurological complications, such as seizures, re-bleeding, hydrocephalus, DCI, and intracranial hypertension, than others. The proportion of patients with hyperlactatemia and hyperglycemia during the 6-day period was higher in those with UO than in those with a favorable outcome (181/209 (87%) vs. 129/247 (52%) *p* = 0.001 and 155/209, 74% vs. 101/247, 41, *p* = 0.001, respectively—Table 2). 

**Figure 2:**
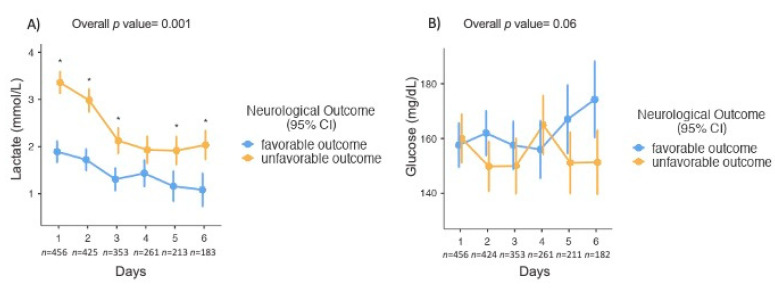
Time course of lactate (**A**) and glucose (**B**) concentrations according to neurological outcome. Lactate and glucose values are expressed as means with 95% confidence intervals. Statistical analyses were conducted using a linear mixed model. * *p* < 0.05 obtained by a post hoc analysis using the Bonferroni method; *n* represents the number of patients included in the analysis for each day.

The time courses of lactate and glucose concentrations during the first 6 days of ICU admission according to neurological outcome are shown in Figure 2. Lactate concentrations in patients with UO were persistently >2.0 mmol/L in the first 48 h after admission, normalizing after day 3; patients with a favorable outcome had normal lactate concentrations throughout the study period (*p* < 0.001). Glucose concentrations were similar in the two groups.

Patients with hyperlactatemia had 3.04 times higher risk of UO than patients with normal lactate levels. After adjusting for the confounders such as age, Fisher scales 3 and 4, hyperglycemia hypotension, and seizures, hyperlactatemia during the first 6 days of hospital stay was associated with a higher chance of UO (OR 4.16, 95% CI 2.52–6.88). Supplemental Electronic Figure S2 illustrates the directed acyclic graph used to perform the adjustment of the multivariable model (Supplemental Electronic Table S2).

## 4. Discussion

In this large cohort of patients with aSAH, hyperlactatemia was a common finding in the early phase after ICU admission: Half of the patients had hyperlactatemia in the first 24 h and about 70% had hyperlactatemia during the first 6 days of admission. Patients with an unfavorable outcome had persistently higher daily lactate levels during the first 6 days after ICU admission than did patients with favorable outcome. Hyperlactatemia in the first 6 days of hospitalization was independently associated with mortality and unfavorable outcome, regardless of the severity of neurological impairment on admission.

High lactate concentrations in patients with SAH may have a multifactorial pathophysiology [30]. These patients often experience a “catecholamine storm” due to hypothalamic and/or brainstem dysfunction [6], which can produce so-called “neurogenic injuries”, such as stunned myocardium and pulmonary edema [31,32], resulting in tissue hypoxia. Hypovolemia can also occur in the early phase after aneurysm rupture [33] due to increased blood pressure and renal perfusion, or as a consequence of the cerebral salt wasting syndrome [34]. Moreover, excessive adrenergic stimulation could also induce increased glucose metabolism, with high production of pyruvate and lactate, the latter being measurable in the systemic circulation. In a later phase, patients with SAH are also susceptible to clinical complications, such as sepsis, which can further increase lactate concentrations [35].

Blood lactate concentrations are therefore commonly elevated after SAH [15,16,18,19], particularly in patients with unfavorable outcomes. Hyperglycemia in the first 6 days after ICU admission was also a common finding in our study. Lactate and glucose concentrations are often interconnected, because glucose metabolism generates lactate though glycolysis [36] and lactate can be a precursor of glucose in the Cori cycle [37]. Additionally, concentrations of both can increase as a “physiological” response to stress [38]. Studies using cerebral microdialysis have shown that lactate concentration can increase in the brain by increased aerobic glycolysis and that lactate can be used as an alternate source of fuel to the brain tissue [13,39]. In the presence of cerebral hypoxia, elevated cerebral lactate and low glucose indicate ischemia and are markers of poor outcome [40].

Blood lactate concentrations have been less studied and have yielded conflicting results: Whereas Poblete et al. [19] reported no association of lactate with neurological recovery, van Donkelaar et al. reported an independent association between lactate concentrations during the first 24 h of ICU admission and an unfavorable outcome after SAH [15], and Aisiku et al. showed that even slightly increased lactate concentrations during the first 24 h after admission were associated with ICU mortality after SAH [16]. Our study showed that hyperlactatemia within the first 6 days of admission was associated with in-hospital mortality and unfavorable outcome at 3 months.

Moreover, we showed that lactate concentrations were highest within the first 24 h of admission, similar to the findings of Okazaki et al. [18], suggesting that the presence of elevated lactate concentrations early after admission could be considered by physicians as a possible “alert sign” for poor outcome in SAH patients. Importantly, the definitions of hyperlactatemia and hyperglycemia we used in this study could be challenged; serum lactate concentrations are rarely >1.0 mmol/L in healthy people and glucose concentrations before meals are <125 mg/dL in patients without diabetes. However, for lactate, we considered the same cut-off that has been used in many studies of sepsis [41] or cardiac arrest patients [42], and the threshold of glucose was that selected in different studies evaluating “standard” glucose control strategies in brain-injured patients [43].

In other clinical situations, such as shock, trauma, sepsis, major surgery, or cardiac arrest, lactate concentrations and lactate kinetics have also been reported to be associated with mortality [35]. In septic shock, lactate concentrations have been used as a target to guide resuscitation strategies [44,45]. However, basing treatment or prognostic decisions on a single measurement is not recommended in clinical practice and an integrated approach using various parameters such as echocardiography, mixed venous saturation, and advanced hemodynamic monitoring is more appropriate and informative [46].

In patients with SAH, hemodynamic monitoring can be used to help guide treatment aimed at optimizing cerebral perfusion pressure and brain oxygenation [47]. Indeed, targeting specific hemodynamic parameters is also important in the treatment of cerebral vasospasm and DCI [48]. Future studies are needed to evaluate whether lactate concentrations could be integrated into this approach on the hemodynamic management of these patients. Moreover, knowing the cause(s) of hyperlactatemia is essential to decide if and how to manage it. Catecholamine storm remains a clinical challenge. In patients with TBI, interesting results have been reported with the use of beta-blockers [49]. Further studies are needed in SAH to assess the potential use of beta-blockers to reduce the sympathetic over stimulation. 

This study had some limitations. First, because of its retrospective design, we had to rely on the information regarding aSAH-related complications from medical charts. Consequently, we were only able to assess outcome in 3 months, since patients undergo routine 3-month evaluations after discharge. Additionally, we chose a long period to study, which may have impacted our results. Second, we did not measure lactate in specific pre-defined time points (i.e. 6, 12 or 24 hafter admission). Therefore, the time point of peak lactate differed between patients; it remains however unknown whether this methodological limitation would affect the validity of our conclusions. Third, we did not compare systemic lactate concentrations with lactate concentrations from cerebrospinal fluid and/or microdialysis, which would have provided additional information about the differences between systemic and cerebral metabolism. Fourth, we did not evaluate the cause of elevated lactate concentrations in this cohort, as this was beyond the scope of the study. Fifth, despite the use of a generalized mixed model to assess the time course of lactate and glucose (i.e., without the need for imputation of missing values), early deaths or ICU discharge might have affected the assessment of lactate and glucose values over time and biased the relationship with patient outcome. Finally, the results are from a single-center cohort, and local practices and policies might limit their generalizability.

## 5. Conclusions

Hyperlactatemia is common in the early phase of aSAH and is associated with hospital mortality and poor neurological outcome. Prospective studies are needed to assess the role of lactate concentrations as a target for hemodynamic management in this patient population.

## Figures and Tables

**Table 1:** Characteristics of the population according to mortality and neurological outcome at 3 months.

	All Patients	FO	UO	*p*-Value	Survivors	Non-Survivors	*p*-Value
	(*n* = 456)	(*n* = 247)	(*n* = 209)		(*n* = 298)	(*n* = 158)	
Age (years), median (IQR)	54 (46–63)	51 (43–59)	56 (48–67)	<0.01	52 (44–60)	56 (47–67)	<0.01
Female, *n* (%)	290 (64)	153 (62)	137 (66)	0.44	187 (63)	103 (64)	0.84
GCS, median (IQR)	13 (3–15)	15 (13–15)	4 (3–12)	<0.01	15 (10–15)	3 (3–9)	<0.01
Fisher 3–4, *n* (%)	419 (94)	145 (60)	184 (89)	<0.01	145 (60)	184 (89)	<0.01
WFNS 4–5, *n* (%)	216 (47)	57 (23)	159 (76)	<0.01	57 (23)	159 (76)	<0.01
SOFA, median (IQR)	5 (2–8)	2 (1–4)	8 (5–10)	<0.01	2 (1–5)	8 (6–10)	<0.01
APACHE II, median (IQR)	13 (8–19)	8 (6–12)	19 (14–22)	<0.01	9 (7–15)	19 (16–22)	<0.01
Comorbidities							
Hypertension, *n* (%)	191 (42)	111 (45)	80 (38)	0.15	134 (45)	57 (36)	0.05
Diabetes, *n* (%)	38 (8)	13 (5)	25 (12)	0.01	22 (7)	16 (10)	0.38
Heart disease, *n* (%)	54 (12)	20 (8)	34 (16)	0.01	27 (9)	27 (17)	0.02
Neurologic disease, *n* (%)	34 (8)	7 (17)	8 (17)	0.72	19 (6)	15 (9)	0.27
Kidney disease, *n* (%)	8 (2)	5 (2)	3 (1)	0.73	6 (2)	2 (1)	0.72
Asthma/COPD, *n* (%)	37 (8)	16 (7)	21 (10)	0.17	18 (6)	19 (12)	0.03
Cancer, *n* (%)	22 (5)	11 (5)	11 (5)	0.83	11 (4)	11 (7)	0.17
Cirrhosis, *n* (%)	5 (1)	1 (0.4)	4 (2)	0.18	1 (0.3)	4 (3)	0.05
Treatments							
Vasopressors, *n* (%)	249 (55)	71 (29)	178 (85)	<0.01	25 (8)	43 (27)	<0.01
Inotropes, *n* (%)	68 (15)	14 (6)	54 (26)	<0.01	113 (38)	136 (85)	<0.01
MV, *n* (%)	268 (59)	77 (31)	192 (92)	<0.01	115 (39)	154 (96)	<0.01
Endovascular coiling, *n* (%)	336 (74)	215 (87)	121 (58)	<0.01	256 (87)	80 (50)	<0.01
Surgical clipping, *n* (%)	72 (16)	29 (12)	43 (21)	0.01	39 (13)	33 (21)	0.04
Complications							
Hypotension *, *n* (%)	356 (78)	199 (81)	157 (75)	0.17	227 (76)	129 (82)	0.19
Rebleeding, *n* (%)	33 (7)	4 (2)	29 (14)	<0.01	8 (3)	25 (16)	<0.01
DCI, *n* (%)	94 (21)	25 (10)	69 (33)	<0.01	45 (15)	49 (31)	<0.01
ICHT, *n* (%)	192 (42)	34 (14)	158 (76)	<0.01	59 (20)	133 (83)	<0.01
Seizures, *n* (%)	110 (24)	46 (19)	64 (31)	<0.01	69 (23)	41 (26)	0.65
Hydrocephalus, *n* (%)	148 (33)	58 (24)	90 (43)	<0.01	81 (27)	42 (67)	<0.01

* in the first 6 days of admission. FO, favorable outcome; UO, unfavorable outcome; WFNS, grade on World Federation of Neurosurgical Societies; SOFA score, Sequential Organ Failure Assessment score; GCS, Glasgow Coma Scale; APACHE score, Acute Physiology and Chronic Health Evaluation score; ICU, intensive care unit; COPD, chronic obstructive pulmonary disease; MV: mechanical ventilation DCI, delayed cerebral ischemia; ICHT, intracranial hypertension; ECMO, extracorporeal membrane oxygenation; SD, standard deviation; IQR, interquartile range. Favorable outcome was defined as a Glasgow Outcome Scale (GOS) of 4–5 and unfavorable outcome as a GOS of 1–3.

**Table 2:** Lactate and glucose concentrations according to prognostic status.

	All Patients	FO	UO	*p*-Value	Survivors	Non-Survivors	*p*-Value
	(*n* = 456)	(*n* = 247)	(*n* = 209)		(*n* = 298)	(*n* = 158)	
Peak lactate concentration, median (IQR)	2.7 (1.8–3.9)	2.1 (1.5–2.9)	3.5 (2.5–4.9)	<0.01	2.3 (1.6–3.0)	3.7 (2.7–5.2)	<0.01
Hyperlactatemia in the first 24h of ICU admission, *n* (%)	234 (51)	85 (34)	149 (71)	<0.01	59 (20)	90 (157)	<0.01
Hyperlactatemia in the first 6 days of ICU stay, *n* (%)	310 (68)	129 (52)	181 (87)	<0.01	171 (57)	139 (88)	<0.01
Highest serum lactate day 1-mmol/L, median (IQR)	2.1 (1.2–3.2)	129 (52)	181 (87)	<0.01	1.7 (1–2.7)	3.1 (2.1–4.4)	<0.01
Highest serum lactate day 2-mmol/L, median (IQR)	1.9 (1.4–2.6)	1.5 (0.9–2.6)	2.9 (1.9–4.1)	<0.01	1.6 (1–2.3)	2.4 (1.8–3.8)	<0.01
Highest serum lactate day 3-mmol/L, median (IQR)	1.4 (1.1–1.8)	1.5 (1–2.1)	2.3 (1.7–3.2)	<0.01	1.2 (0.9–1.6)	1.7 (1.3–2.1)	<0.01
Highest serum lactate day 4-mmol/L, median (IQR)	1.3 (1.0–1.8)	1.1 (0.9–1.6)	1.6 (1.2–2)	<0.01	1.2 (0.9–1.5)	1.5 (1.1–2.1)	<0.01
Highest serum lactate day 5-mmol/L, median (IQR)	1.2 (0.9–1.6)	1.2 (0.9–1.5)	1.4 (1.1–2)	<0.01	1.1 (0.9–1.5)	1.3 (1.1–1.8)	<0.01
Highest serum lactate day 6-mmol/L, median (IQR)	1.2 (0.9–1.5)	1.1 (0.8–1.3)	1.4 (1.1–1.8)	<0.01	1.1 (0.9–1.4)	1.3 (1–1.7)	<0.01
Peak glucose-mg/dL, median (IQR)	188 (157–231)	171(146-204)	214 (180-265)	<0.01	176 (149–210)	218 (180–267)	<0.01
Hyperglycemia in the first 24 h of ICU admission, *n* (%)	121 (27)	33 (13)	88 (42)	<0.01	46 (15)	75 (48)	<0.01
Hyperglycemia in the first 6 days of ICU admission, *n* (%)	256 (56)	101 (41)	155 (74)	<0.01	248 (83)	155 (98)	<0.01
Highest blood glucose day 1-mg/dL, median (IQR)	157 (131–188)	149 (123–176)	167 (142–203)	<0.01	150 (125–181)	171 (148–206)	<0.01
Highest blood glucose day 2-mg/dL, median (IQR)	154 (133–183)	149 (127–166)	163 (138–193)	<0.01	151 (131–177)	163 (139–198)	<0.01
Highest blood glucose day 3-mg/dL, median (IQR)	148 (126–167)	148 (127–163)	149 (126–172)	<0.01	149 (127–167)	143 (123–165)	<0.01
Highest blood glucose day 4-mg/dL, median (IQR)	149 (130–188)	147 (126–189)	156 (134–187)	0.03	148 (127–189)	155 (137–187)	<0.12
Highest blood glucose day 5-mg/dL, median (IQR)	152 (129–186)	151(123–174)	156 (137–192)	<0.01	151 (127–181)	160 (138–192)	0.02
Highest blood glucose day 6-mg/dL, median (IQR)	149 (128–176)	143 (126–170)	156 (129–179)	0.27	148 (127–176)	150 (129–178)	0.93

IQR, interquartile range. Hyperlactatemia was defined as lactate > 2.0 mmol/L. Hyperglycemia was defined as a serum glucose >180 mg/dL.

## Supplementary Materials

The following are available online at https://www.mdpi.com/2076-3425/10/12/1004/s1, Figure S1. Directed acyclic graph for hospital mortality. This graph illustrates the causal relationship between hyperlactatemia in the first 6 days of hospital admission and in-hospital mortality (death). Variables in gray represent adjusted variables. Circular nodes represent unobserved variables and rectangular nodes represent other variables. Figure S2. Directed acyclic graph for poor neurological outcome in 3 months. This graph illustrates the causal relationship between hyperlactatemia in the first 6 days of hospital admission and unfavorable neurological outcome in 3 months assessed by the Glasgow outcome scale. Variables in gray represent adjusted variables. Circular nodes represent unobserved variables and rectangular nodes represent other variables. Table S1. Logistic regression model for factor related to mortality. Table S2. Logistic regression model for factors related to unfavorable neurological outcome in 3 months.
