# Supplementary material for: The Prognostic Role of Lactate Concentrations after Aneurysmal Subarachnoid Hemorrhage
Source: Brain Sci. 2020 Dec 17;10(12):1004. doi: 10.3390/brainsci10121004 (PMC7766816; doi:10.3390/brainsci10121004)
Supplement: Supplementary file 1 [file brainsci-10-01004-s001.pdf]

Table S1. Logistic regression analysis for hospital mortality.

	Univariate analysis<br>OR [CI 95%]	Multivariable analysis<br>OR [CI 95%]
Age	1.03 [1.02-1.05]	1.02 [1.01-1.04]
Fisher 3-4	7.29 [1.70-31.21]	3.41 [0.78-14.85]
Cirrhosis	7.71 [0.86-69.62]	10.50 [0.99-110.45
Hypotension	1.39 [0.86-2.26]	1.56 [0.90-2.68]
Hyperlactatemia	5.43 [3.19-9.25]	4.19 [2.38-7.39]
Hyperglycemia	10.41 [3.19-33.97]	2.46 [1.56-3.90]
Seizures	1.16 [0.74-1.82]	0.91 [0.56-1.05]

This model was constructed to assess causal association between hyperlactatemia and in-hospital mortality according to the directed acyclic graph presented on Fig S1. All other variables are confounders. Hyperlactatemia was defined as lactate >2.0 mmol/L at any time during the first 6 days of admission. Hyperglycemia was defined as a serum glucose >180 mg/dL at any time during the first 6 days of admission.

Table S2. Univariate and multivariable analysis of unfavorable neurological outcome.

	Univariate analysis<br>OR [CI 95%]	Multivariable analysis<br>OR [CI 95%]
Age	1.04 [1.02-1.06]	1.03 [1.02-1.05]
Fisher 3-4	5.31 [3.20-8.79]	4.04 [1.02-15.92]

Seizures	0.53 [0.34-0.81]	1.42 [0.86-2.33]
Hypotension	0.73 [0.47-1.14]	0.78 [0.47-1.31]
Hyperlactatemia	5.91 [3.70-9.46]	4.16 [2.52-6.88]
Hyperglycemia	4.15 [2.78-6.19]	2.82 [1.82-4.34]

This model was constructed to assess causal association between hyperlactatemia and unfavorable outcome in 3 months, according to the directed acyclic graph presented on Fig S2. All other variables are confounders. Hyperlactatemia was defined as lactate >2.0 mmol/L at any point during the first 6 days of admission. Hyperglycemia was defined as a serum glucose >180 mg/dL at any point during the first 6 days of ICU stay.

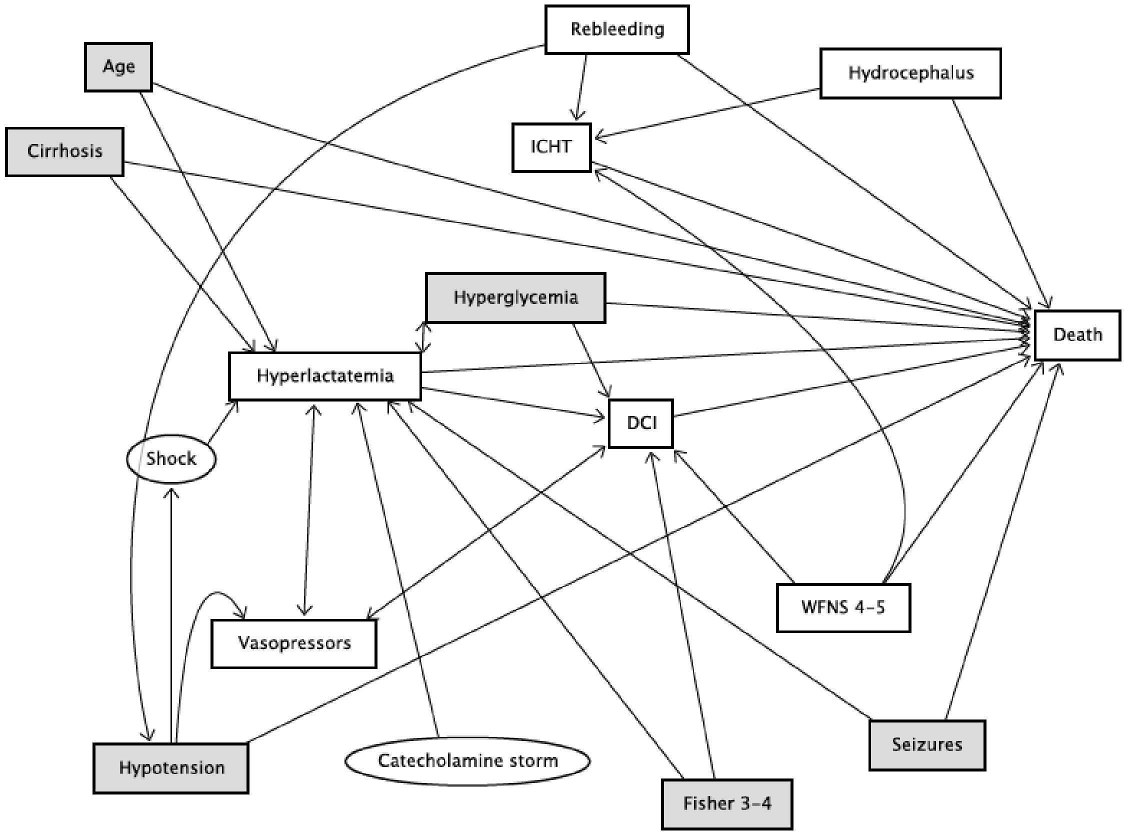

Figure S1. Directed acyclic graph for hospital mortality. This graph illustrates the causal relationship between hyperlactatemia in the first 6 days of hospital admission and in-hospital mortality (death). Variables in gray represent adjusted variables. Circular nodes represent unobserved variables and rectangular nodes represent other variables.

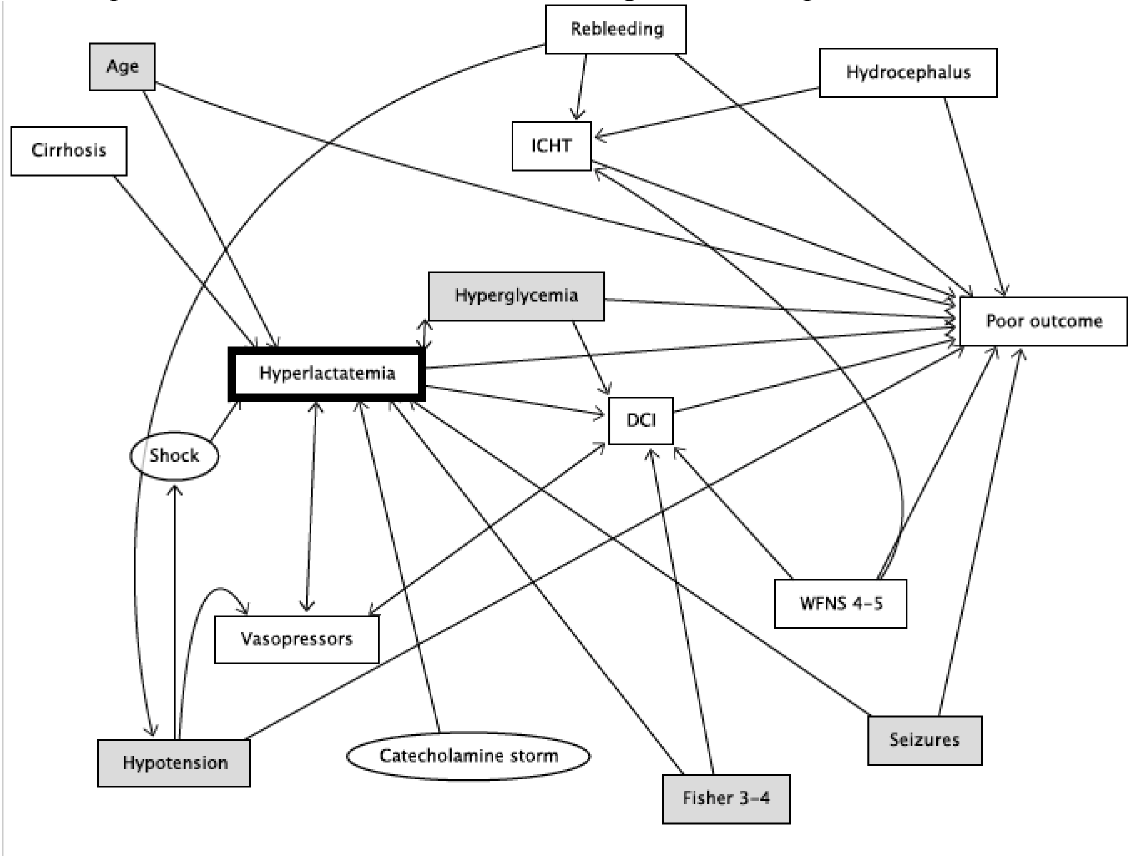

Figure S2. Directed acyclic graph for poor neurological outcome in 3 months. This graph illustrates the causal relationship between hyperlactatemia in the first 6 days of hospital admission and unfavorable neurological outcome in 3 months assessed by the Glasgow outcome scale. Variables in gray represent adjusted variables. Circular nodes represent unobserved variables and rectangular nodes represent other variables.
